# 

**Published:** 1908-07-15

**Authors:** 


CONTENTS

Event and Comment - - - - - - - - 325
Oral Prophylaxis, - *By Mabel M. Bennett, A. B., D.D.S.* 331
Trials and Tribulations of Dentists Fifty Years Ago, *By Dr. Charles H. Bartlett.* 352
Some Things for Dentists to Think About, *By S. E. Dodson, D.D.S.* 359
Quick Control of Pain and Disease without Drugs by Use of the Leucodescent Light, - *By F. E. Raiche, D.D.S.* 364
Indents - - - - - - - - - - - 369
Announcements - - - - - - - - - 376

